# A 16S rRNA-Based Screening Framework Reveals Distinct Microbial and Antimicrobial Resistance-Related Fingerprints in Aquaculture and Municipal Wastewater Effluents

**DOI:** 10.3390/toxics14100852

**Published:** 2026-09-24

**Authors:** Junjie Meng, Yun Liu, Rongpeng Dou, Shuhan Yu, Shengwang Gao, Yuxuan Zhang, Qingjia Meng

**Affiliations:** 1 College of Bioscience and Resources Environment, Beijing University of Agriculture, Beijing 102206, China; 2 Chinese Research Academy of Environmental Sciences, Beijing 100012, China; 3 Linqu Sub-Bureau of Weifang Municipal Ecology and Environment Bureau, Weifang 261000, China

**Keywords:** reclaimed water, opportunistic pathogens, antibiotic resistance genes, aquaculture, wastewater treatment plant, ecological risk assessment

## Abstract

Bacterial taxa of potential public-health relevance and predicted antimicrobial-resistance functions are important in effluent management. This study compared bacterial communities, environmental associations, and predicted functions in aquaculture effluents (Group A) and wastewater treatment plant effluents (Group W) from the Mi River Basin, China, using a 16S rRNA-based framework, with the WHO 2024 Bacterial Priority Pathogens List used for contextual interpretation. relatively consistent profiles, with *Acinetobacter*, *Limnohabitans*, and *Pseudomonas* among abundant genera. Group W showed site-specific patterns, including high relative abundances of *Legionella*, *Flavobacterium*, and *Mycobacterium* in W3 and *Pseudarcobacter* and *Arcobacter* in W4 and W5. Among 400 genus–factor correlations, only the negative *Sphingorhabdus*–COD association remained significant after BH correction. The aggregate predicted abundance of six ARG-related KOs was 2.22-fold higher in Group A. The *tetA*-type KO was higher in Group A, whereas *erm*-type was higher in Group W. Seven samples had abundance-weighted NSTI values below 0.10, and directional patterns remained after excluding W5. Although 16S-based analyses cannot directly confirm pathogens or ARGs, the framework distinguished effluent-specific microbial and predicted resistance-related patterns, supporting large-scale routine monitoring and tiered risk management for water reuse and receiving waters.

## 1. Introduction

Water scarcity and declining water quality are major constraints on global sustainable development. By 2050, the global urban population facing water scarcity is projected to reach 1.7–2.4 billion [1]. Wastewater generation is also expected to increase substantially with continued urbanization [2]. Water reclamation and reuse are important strategies for alleviating water stress and promoting a circular economy. Globally, only about 11% of wastewater is intentionally reused, mainly for irrigation [3]. However, high nitrogen and phosphorus loads and microbial contaminants, including opportunistic pathogens and antibiotic-resistant bacteria (ARB), continue to limit the safe reuse of reclaimed water [4]. Aquaculture systems and wastewater treatment plants (WWTPs) are two distinct sources of reclaimed-water effluent considered in this study.

Beyond conventional pollutants, antibiotic resistance genes (ARGs) and opportunistic pathogens are increasingly recognized as emerging contaminants of concern in water reuse. ARGs can proliferate with their bacterial hosts and spread through horizontal gene transfer, and opportunistic pathogens can persist and disseminate across environmental compartments. After entering downstream aquatic ecosystems, these contaminants may cause prolonged exposure in fish, benthic organisms, and waterbirds. They may also pose potential risks to human health through the food chain or direct contact [5,6]. Emerging and conventional contaminants may be closely coupled during their occurrence, transport, and transformation. Organic loads and nutrient levels may influence the community structure of opportunistic pathogens and ARG-hosting bacteria. In turn, these microbial communities may contribute to the transformation and biogeochemical cycling of conventional contaminants [7,8,9].

China’s aquaculture output approached 50 million tonnes in 2018. Pollutant discharge per tonne of aquaculture product reached 13.6 kg of chemical oxygen demand (COD), 0.45 kg of ammonia nitrogen (NH_3_-N), 2.02 kg of total nitrogen (TN), and 0.33 kg of total phosphorus (TP) [10]. Intensive aquaculture commonly uses antibiotics for disease control, and antibiotic residues may exert selective pressure on microbial communities. A survey of 40 freshwater fish ponds near Wuxi, China, detected enrofloxacin at concentrations up to 2.36 μg/L and sulfadiazine at concentrations exceeding 4 μg/L [11]. These conditions may favor the occurrence and persistence of ARGs and ARB in aquaculture waters. Sulfonamide- and tetracycline-resistance genes are frequently reported as the predominant ARG types [12]. Studies of coastal aquaculture systems have also reported the dissemination of multidrug resistance [13]. ARGs may persist across successive stages of aquaculture production [14]. Opportunistic bacterial genera found in aquaculture ponds, including *Acinetobacter* and *Pseudomonas*, may also serve as important potential hosts of ARGs [15]. Aquaculture effluent may introduce potentially pathogenic bacteria and antimicrobial-resistance determinants into receiving waters [16].

Although WWTP effluent undergoes secondary biological treatment, it may still introduce chemical pollutants and microbial contaminants into receiving waters. A study of nine WWTPs in Japan and Thailand detected *Aeromonas*, *Arcobacter*, and *Mycobacterium* in all influent and effluent samples. In particular, *Mycobacterium* was not effectively removed, which may suggest selective enrichment of potentially pathogenic bacteria during the treatment process [17]. Conventional WWTP processes may also incompletely remove ARB and ARGs. Their continued release may facilitate the proliferation, transfer, and dissemination of antimicrobial resistance [18,19]. In three Italian WWTPs, *Arcobacter*, *Acinetobacter*, *Flavobacterium*, and *Pseudarcobacter* were the dominant genera [19]. Notably, *Acinetobacter* includes carbapenem-resistant *A. baumannii*, a critical-priority pathogen in the WHO 2024 Bacterial Priority Pathogens List (BPPL). WWTP effluent commonly carries ARGs such as *qnrS*, *sul1*, *tetW*, and *ermB* [20]. Even when conventional water-quality parameters meet discharge standards, secondary effluent may remain an important route for disseminating opportunistic pathogens and ARGs into downstream waters.

Given the potential value of wastewater from aquaculture and WWTPs as reclaimed water, this study conducted a systematic comparative investigation within a unified watershed. Eight water-quality parameters were measured, and bacterial communities were characterized using high-throughput sequencing. Functional Annotation of Prokaryotic Taxa (FAPROTAX) and Phylogenetic Investigation of Communities by Reconstruction of Unobserved States 2 (PICRUSt2) were used for functional assignment and prediction. The study had three objectives. First, we compared conventional water quality and bacterial community composition between the two effluent types. Second, we evaluated associations between environmental factors and community differentiation. Third, we characterized genera of potential public-health relevance and predicted antimicrobial-resistance-related functional potential. External concordance was assessed using published qPCR, SmartChip, and metagenomic datasets. The findings provide a basis for differentiated effluent monitoring and targeted confirmatory analysis.

## 2. Materials and Methods

### 2.1. Study Area and Sample Collection

**Figure 1:**
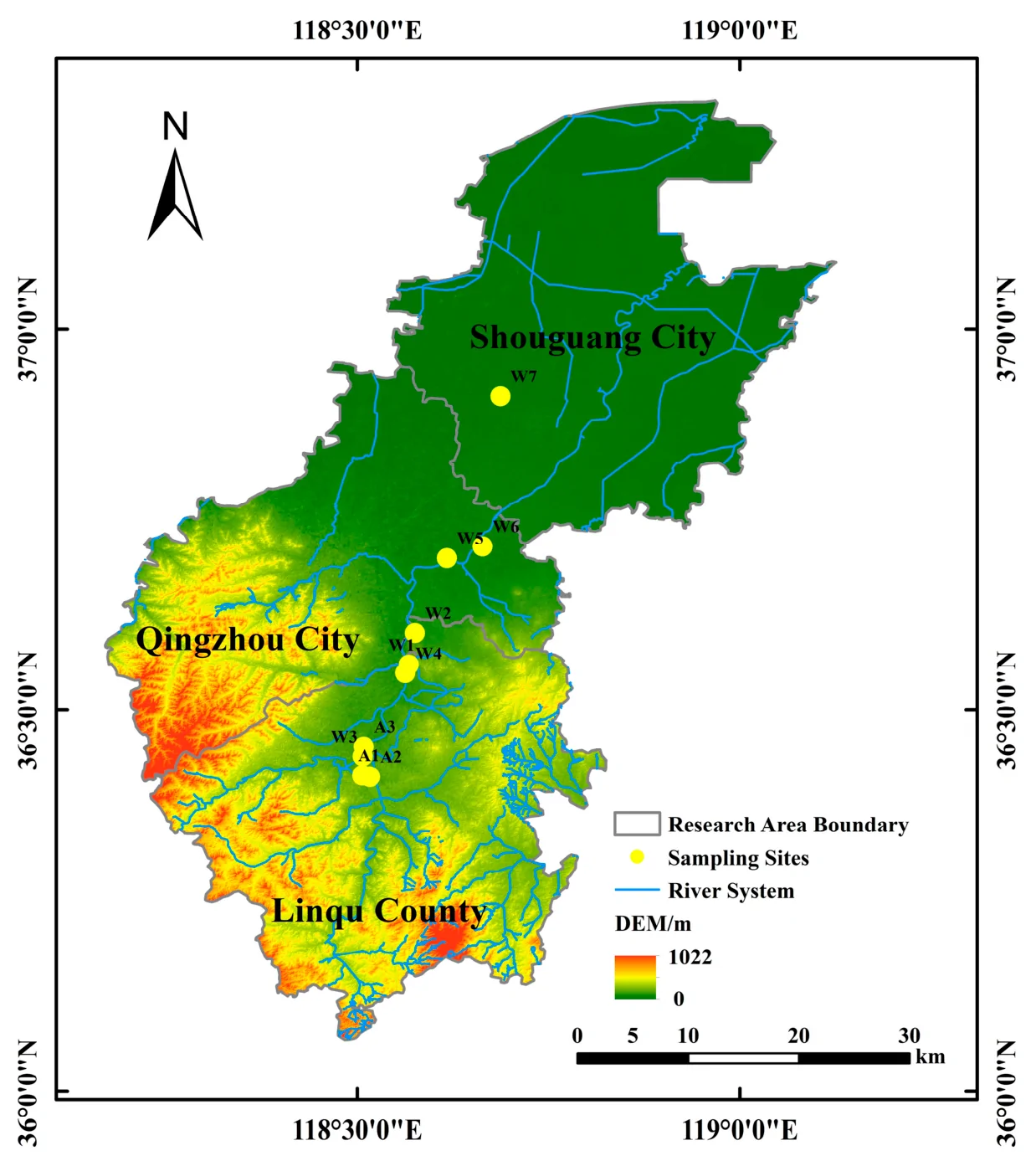
Overview of the study area and distribution of sampling sites.

The study area is located in Weifang City, Shandong Province, China (Figure 1). The terrain generally slopes from south to north and from west to east. The area has a dense river network and represents a typical transition from mountainous terrain to plains. Numerous aquaculture ponds and wastewater treatment plants (WWTPs) are distributed throughout the region. Both aquaculture effluent and WWTP effluent are ultimately discharged into Laizhou Bay via the Mi River. These characteristics provide a suitable setting for comparing bacterial community patterns in aquaculture and WWTP effluents discharged to a common receiving-water system.

A total of 10 independent grab samples were collected between 1 and 17 March 2024 from three regional aquaculture-effluent discharge sites (A1–A3; Group A) and seven WWTP final-effluent or discharge sites (W1–W7; Group W). The aquaculture zones in the Mi River basin are primarily concentrated in the upper reaches, with sturgeon farming being the dominant activity. The water sources, farming practices, and drainage systems for these operations are relatively stable, and the farming cycles follow a seasonal pattern. Therefore, we conducted sampling exclusively during individual seasons. The three sampling points selected are representative of the study area. The seven Group W sites were selected as representative final-effluent or discharge outlets of the WWTPs. Under routine operating conditions, their treated effluent was considered relatively stable. At each sampling point, three replicate samples were collected, pooled, and then subjected to subsequent analysis without temporal replication. Future studies will consider incorporating repeated sampling across seasons and operating conditions. Site coordinates, sampling dates, wastewater sources, and available WWTP information are provided in Supplementary Table S1A. Each sample was collected in a sterile polyethylene bottle and divided into two portions. One portion was refrigerated for laboratory analyses of COD, TN, TP, and NH_3_-N, whereas the other was filtered and stored at −80 °C before microbial analysis.

### 2.2. Analytical Methods

Water temperature (T), salinity (Sal), dissolved oxygen (DO), and pH were measured in situ following standard water-quality monitoring procedures. Chemical oxygen demand (COD) was determined using the dichromate method. Total nitrogen (TN) was measured using alkaline potassium persulfate digestion followed by ultraviolet spectrophotometry. Total phosphorus (TP) was determined using the ammonium molybdate spectrophotometric method. Ammonia nitrogen (NH_3_-N) was measured using Nessler’s reagent spectrophotometry. DNA extraction and high-throughput sequencing of bacterial 16S rRNA gene amplicons were performed by Shanghai Majorbio Bio-Pharm Technology Co., Ltd. (Shanghai, China). Total microbial genomic DNA was extracted using the E.Z.N.A.^®^ Soil DNA Kit (Omega Bio-tek, Norcross, GA, USA) according to the manufacturer’s instructions. The V3–V4 region of the bacterial 16S rRNA gene was amplified using primers 338F (5′-ACTCCTACGGGAGGCAGCAG-3′) and 806R (5′-GGACTACHVGGGTWTCTAAT-3′) with TransStart^®^ FastPfu DNA Polymerase (TransGen Biotech Co., Ltd., Beijing, China). The resulting amplicons were subjected to 300-bp paired-end sequencing on an Illumina NextSeq 2000 platform (Illumina, Inc., San Diego, CA, USA).

### 2.3. Bioinformatics and Statistical Analysis

Raw paired-end reads were quality-filtered using fastp v0.19.6 and merged using FLASH v1.2.11. Bacterial 16S rRNA gene sequences were clustered into operational taxonomic units (OTUs) at 97% similarity using UPARSE v7.1, with chimeric sequences removed. Taxonomy was assigned using the RDP Classifier v2.11 against the SILVA v138 database at a 70% confidence threshold. Functional profiles and predicted abundances were generated using PICRUSt2 v2.2.0_b and the Kyoto Encyclopedia of Genes and Genomes (KEGG) database [21] on the Majorbio Cloud Platform. KEGG Level 2 and selected Level 3 pathways were analyzed. Six ARG-related KOs were selected according to KEGG annotations and relevant literature. Abundance-weighted nearest sequenced taxon index (NSTI) values were calculated to assess reference-genome support for functional prediction. Lower NSTI values indicated closer phylogenetic relationships between the observed OTUs and sequenced reference genomes.

External concordance of six ARG-related KOs (K18974, K18824, K08151, K18220, K18555, and K00561) was evaluated using three published qPCR, SmartChip, and metagenomic datasets [22,23,24]. Concordance was classified at the gene, ARG-family, or resistance-mechanism level. These external datasets provided contextual evidence only. FAPROTAX v1.2.12 was applied to genus-level profiles to examine six pathogen- and host-associated functional categories. PICRUSt2 and FAPROTAX outputs were treated as inferred, hypothesis-generating evidence and interpreted alongside the observed community composition. The complete PICRUSt2-predicted KO abundance matrix is provided in Table S9. Sensitivity analyses excluded W5 from PICRUSt2 comparisons because of its high NSTI and W3 from FAPROTAX comparisons to assess single-sample influence.

Group differences were tested using two-sided Wilcoxon rank-sum tests. Non-metric multidimensional scaling (NMDS) was based on Bray–Curtis dissimilarities. Permutational multivariate analysis of variance (PERMANOVA) and Permutational analysis of multivariate dispersions (PERMDISP) used 9999 permutations to assess group separation and homogeneity of dispersion, respectively. Canonical correspondence analysis (CCA) and Spearman correlation analyses were used to evaluate relationships among bacterial communities, individual genera, and water-quality parameters. For CCA, inertia, axis significance, and variance inflation factors were calculated. Because the CCA included eight water-quality variables for 10 samples, it was interpreted as exploratory. Benjamini–Hochberg correction was applied within each predefined family of multiple comparisons, and both raw and adjusted *p* values were reported. Cliff’s delta was calculated for water-quality variables and the six ARG-related KOs. All analyses and visualizations were performed in R v4.5.0 (R Foundation for Statistical Computing, Vienna, Austria), with community analyses implemented using the vegan package v2.7.3. A two-sided *p* < 0.05 was considered significant. For multiple comparisons, significance was determined using BH-adjusted *p* values.

## 3. Results

### 3.1. Physicochemical Water-Quality Characteristics

Eight measured water-quality parameters for aquaculture effluent (Group A) and WWTP effluent (Group W) are summarized in Table 1. Wilcoxon rank-sum tests yielded raw *p* values of 0.017 for Sal and COD. However, neither difference remained significant after Benjamini–Hochberg (BH) correction (adjusted *p* = 0.067). Mean salinity was 2631.43 ppm in Group W and 460.33 ppm in Group A, representing an approximately 5.7-fold difference. Salinity varied more widely in Group W (SD = 1606.74 ppm) than in Group A (SD = 196.41 ppm). Four WWTPs (W1, W4, W5, and W6) received wastewater from industrial parks. Differences in influent composition may have contributed to the higher salinity and greater variation among WWTP sites. Mean COD was also higher in Group W than in Group A (33.71 vs. 11.33 mg/L). These WWTPs used different treatment processes, including anaerobic–anoxic–oxic (A^2^/O) treatment, ozonation, and activated-coke adsorption. This process heterogeneity may have contributed to the observed variation in COD among Group W samples.

TN showed a higher mean concentration and greater variation in Group A than in Group W. This pattern may be associated with differences in feed inputs, residual feed decomposition, and nutrient accumulation among aquaculture systems. Mean TP was also higher in Group A than in Group W (0.28 vs. 0.15 mg/L). This numerical pattern may reflect phosphorus inputs and cycling associated with aquaculture activities [25]. In contrast, mean NH_3_-N was slightly lower in Group A than in Group W (0.67 vs. 0.90 mg/L). Together with the TN results, this pattern may indicate differences in nitrogen composition between the two effluent types. Mean DO was lower in Group A, which may be related to biological oxygen consumption, organic-matter decomposition, or diurnal fluctuations in aquaculture systems [26].

### 3.2. Microbial Community and Diversity

#### 3.2.1. Microbial Community Diversity

**Figure 2:**
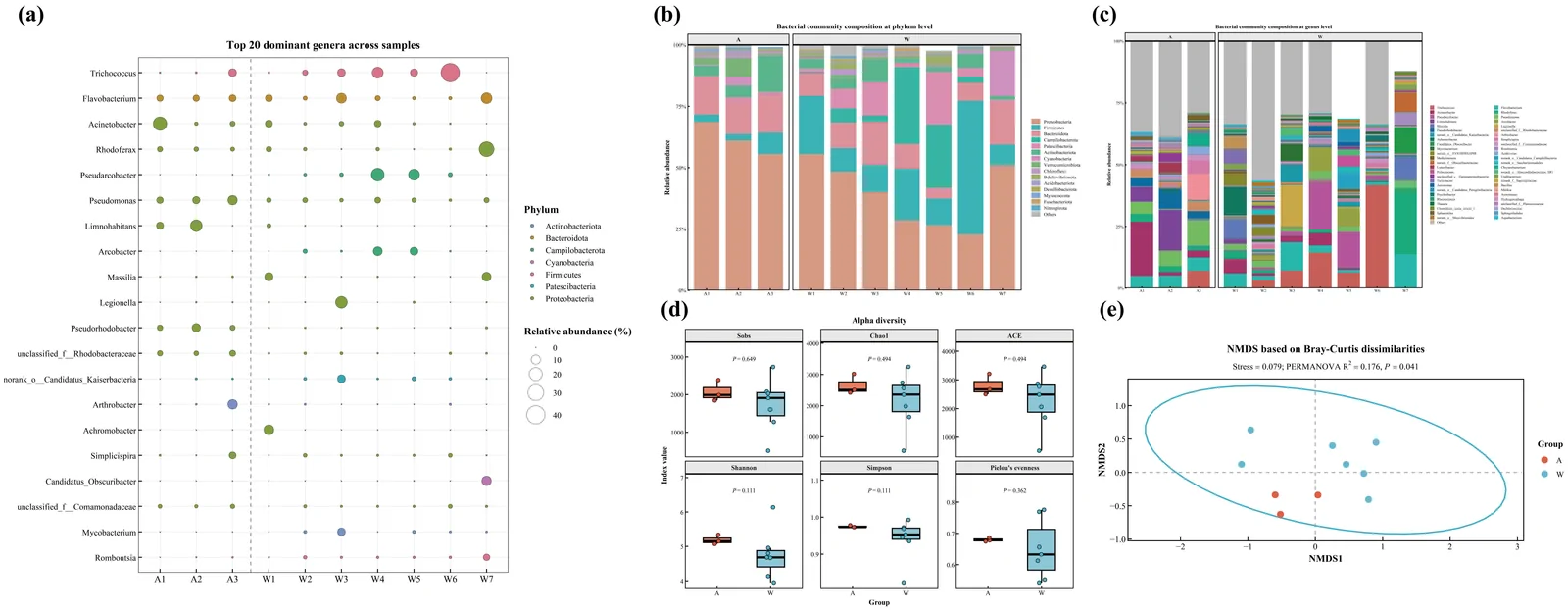
Microbial communities and diversity of two effluent types. (**a**) Bubble plot showing the relative abundances of the 20 most abundant genera across the ten samples; (**b**) Relative abundance of bacterial communities at the phylum-level; (**c**) Relative abundance of bacterial communities at the genus level; (**d**) Box plots of six alpha-diversity indices. Between-group differences were evaluated using two-sided Wilcoxon rank-sum tests. The displayed *p* values are unadjusted, and the BH-adjusted values are provided in Table S2; (**e**) Two-dimensional non-metric multidimensional scaling (NMDS) ordination based on Bray–Curtis dissimilarities. Ellipses represent 95% confidence intervals. Group separation and homogeneity of dispersion were evaluated using PERMANOVA and PERMDISP, respectively, with 9999 permutations. Detailed PERMANOVA and PERMDISP results are provided in Table S3. In (**a**), the vertical dashed line separates Group A (A1–A3) from Group W (W1–W7). In (**d**), the overlaid points represent individual samples.

After rarefaction, alpha diversity was assessed using six indices (Figure 2d). Good’s coverage was ≥0.980 for all samples, indicating adequate sequencing depth. Group A showed slightly higher mean richness and diversity, with more consistent values among samples. However, none of the six indices differed significantly between the groups (all raw *p* ≥ 0.111; all BH-adjusted *p* ≥ 0.332). The relatively consistent values in Group A may reflect similar pond-management practices. Variation among Group W samples may be related to differences in influent sources, treatment processes, and operating conditions. NMDS based on Bray–Curtis dissimilarities indicated separation between the two groups (stress = 0.079; Figure 2e). Group A clustered closely, whereas Group W was more widely dispersed and had a larger 95% confidence ellipse. PERMANOVA detected a significant difference in community composition between the two effluent types (R^2^ = 0.176, *p* = 0.041). Group identity explained 17.6% of the total variation. However, PERMDISP did not detect a significant difference in within-group dispersion (*p* = 0.202). Overall, the two effluent types differed in bacterial community composition, but the greater dispersion observed in Group W was not statistically significant.

#### 3.2.2. Phylum-Level Community

A total of 54 bacterial phyla and candidate phyla were detected after the removal of chloroplast and mitochondrial sequences. Across all samples, Proteobacteria (45.9%), Firmicutes (15.3%), Bacteroidota (12.0%), and Campilobacterota (6.8%) were the predominant phyla (Figure 2a,b). Group A showed relatively consistent community structures. Proteobacteria was the dominant phylum in Group A (61.8%), followed by Bacteroidota (14.5%) and Actinobacteriota (7.9%). A2 contained relatively high abundances of Cyanobacteria (3.7%) and Verrucomicrobiota (7.5%), which have been associated with nutrient-rich environments [27]. By contrast, Group W showed substantial sample-specific variation. The mean relative abundance of Proteobacteria was lower in Group W than in Group A (39.0% vs. 61.8%; raw *p* = 0.023). However, the difference did not remain significant after BH correction (adjusted *p* = 0.724).

Firmicutes, Cyanobacteria, Campilobacterota, and Patescibacteria showed higher mean relative abundances in Group W (Table S1B). Firmicutes and Campilobacterota each had a raw *p* value of 0.040, but neither remained significant after BH correction (adjusted *p* = 0.724). Campilobacterota was nearly absent from Group A but reached 31.3% in W4 and 25.8% in W5. This phylum was mainly represented by *Arcobacter* and *Pseudarcobacter*. Both genera have been associated with wastewater or enteric sources, and some *Arcobacter* species are considered potential zoonotic and foodborne pathogens [17,28,29]. After chloroplast sequences were removed, true Cyanobacteria reached 18.5% in W7 but remained below 4% in the other samples. This pattern may reflect the proliferation and discharge of photosynthetic bacteria from sedimentation tanks [30]. Firmicutes reached 54.4% in W6, mainly because of the enrichment of *Trichococcus*. Patescibacteria also showed sample-specific enrichment, reaching 8.0%, 13.5%, and 21.6% in W2, W3, and W5, respectively. Members of this phylum commonly have small genomes and host-dependent lifestyles [31].

#### 3.2.3. Genus-Level Community

Group A and Group W showed distinct community patterns, with greater sample-specific variation in Group W (Figure 2c). Across all samples, *Trichococcus* (8.1%) and *Flavobacterium* (5.3%) had the highest mean relative abundances. Other abundant genera included *Acinetobacter*, *Rhodoferax*, *Pseudarcobacter*, *Pseudomonas*, and *Limnohabitans*. However, their distributions varied considerably among samples.

Group A showed a relatively consistent community structure, although some genera exhibited sample-specific enrichment. The abundant genera included *Acinetobacter* (mean, 8.7%), *Limnohabitans* (mean, 7.5%), *Pseudomonas* (mean, 7.2%), and *Flavobacterium* (mean, 5.0%). *Flavobacterium* was relatively evenly distributed among the three samples. *Acinetobacter* reached 22.0% in A1 but remained below 3% in A2 and A3. This genus includes clinically important species such as *A. baumannii*, but species-level validation would be required [32,33]. *Arthrobacter* also showed a concentrated distribution, reaching 10.6% in A3.

Group W showed pronounced sample-specific variation, with almost every sample characterized by a distinct dominant taxon (Table 2). *Trichococcus* dominated W6 (41.7%) and was also abundant in W4 (14.4%) and W5 (6.4%). This distribution contributed to the high relative abundance of Firmicutes in these samples. W1 was characterized by *Achromobacter* (11.5%), together with relatively high abundances of *Massilia*, *Acinetobacter*, *Turicibacter*, *Flavobacterium*, and *Bacillus*. W7 was characterized by *Rhodoferax* (26.7%), *Flavobacterium* (13.7%), and Candidatus Obscuribacter (10.5%).

*Pseudarcobacter* and *Arcobacter* were abundant in W4 and W5. These enteric-associated genera are frequently detected in animal feces and sewage systems [17,34,35]. W4 mainly treats domestic sewage from the northern urban area and industrial wastewater from the economic development zone. W5 serves four towns and an industrial park. Human- and livestock-derived fecal inputs may provide continuous sources of these enteric-associated bacteria. Both plants also use the A^2^/O process. Its alternating anaerobic, anoxic, and oxic conditions may favor the persistence of facultatively anaerobic Campilobacterota members in the effluent. *Legionella* reached 16.7% in W3 but remained below 1% in the other samples. *Mycobacterium* also reached 7.0% in W3. Both genera include opportunistic pathogens and may persist in engineered water systems [36,37]. The “others” category accounted for 56.6% of W2, and no individual genus exceeded 5%. This pattern indicated a community composed of numerous low-abundance taxa. The W2 influent mainly originates from a high-tech industrial park and is collected through the municipal sewer network. The complex composition and variable biodegradability of industrial wastewater may hinder the establishment of a single dominant taxon.

Overall, the genus-level patterns supported the phylum-level results. Firmicutes enrichment was mainly associated with *Trichococcus*, whereas Campilobacterota enrichment was associated with *Pseudarcobacter* and *Arcobacter*. These patterns identified several dominant or sample-specific genera of potential ecological or public-health interest.

### 3.3. Environmental Associations of Microbial Community Structure

**Figure 3:**
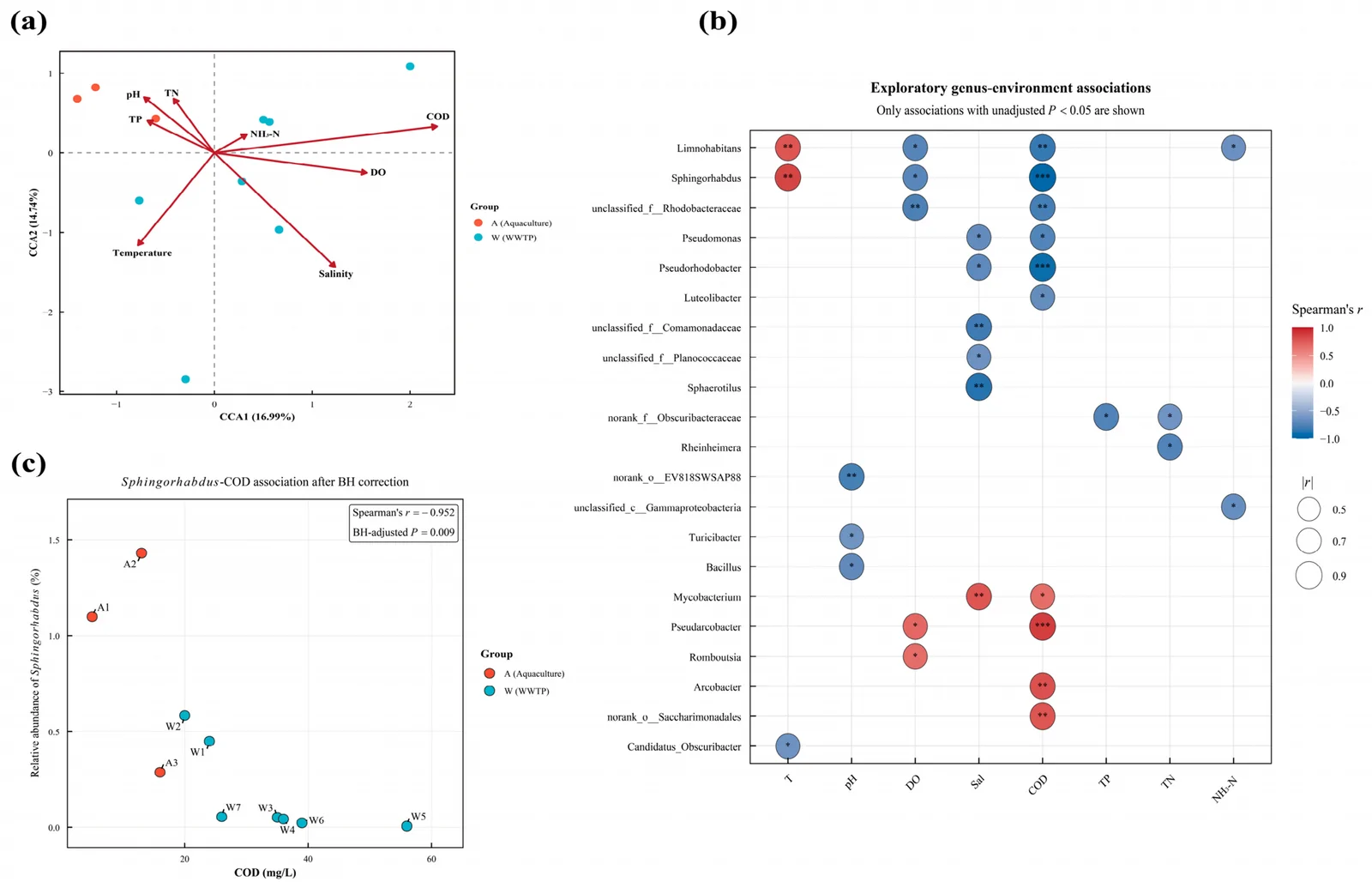
Associations between environmental factors and microbial community structure in the two effluent types. (**a**) Exploratory canonical correspondence analysis (CCA) constrained by eight environmental variables. Detailed inertia, permutation-test, and variance inflation factor results are provided in Table S4A–C; (**b**) Bubble plot showing the 32 genus–factor correlations with unadjusted *p* < 0.05 among the top 50 dominant genera and eight environmental factors, with asterisks indicating significance levels (* *p* < 0.05, ** *p* < 0.01, *** *p* < 0.001); (**c**) Relationship between COD concentration and the relative abundance of *Sphingorhabdus* across the ten samples.

Exploratory CCA suggested three environmental association patterns. Sal, DO, and COD were most clearly associated with between-group separation. TN and TP were associated mainly with secondary variation. T, pH, and NH3-N showed relatively weak relationships with community composition. The eight environmental variables accounted for 88.1% of the total inertia. However, the overall CCA model was not significant (*p* = 0.803). CCA1 and CCA2 explained 17.0% and 14.7% of the constrained inertia, respectively (31.7% jointly; Figure 3a). No individual CCA axis was significant (all *p* = 0.801). Variance inflation factors ranged from 1.90 to 8.21, with none exceeding 10. Group A samples clustered mainly in the negative region of CCA1, whereas Group W samples were more dispersed across the central and positive regions. The positions of Group W samples were visually associated with COD and DO. Sal was also associated with variation along CCA2. TN and TP showed similar orientations toward Group A, suggesting similar associations with the ordination space. Because the model included eight environmental variables for only 10 samples, the CCA results of this study were interpreted as exploratory.

Spearman analysis identified 32 genus–factor correlations with unadjusted *p* < 0.05 (Figure 3b). The complete correlation results, including raw and BH-adjusted *p* values, are provided in Table S5. After BH correction across 400 comparisons, only the negative correlation between *Sphingorhabdus* and COD remained significant (adjusted *p* = 0.009; Figure 3c). *Sphingorhabdus* was relatively enriched in the low-COD Group A samples. This association may partly reflect between-group differences in both COD and relative abundance. Before correction, COD, DO, and Sal showed more genus-level associations than the other environmental factors. Correlations involving individual genera and NH3-N, TN, TP, or pH did not remain significant after correction. The unadjusted associations were therefore interpreted descriptively. Overall, the exploratory analyses suggested that Sal, DO, and COD were most closely associated with community differentiation. COD showed the strongest genus-specific association.

### 3.4. Functional Prediction of Microbial Community

#### 3.4.1. Overall Predicted Functional Profiles

**Figure 4:**
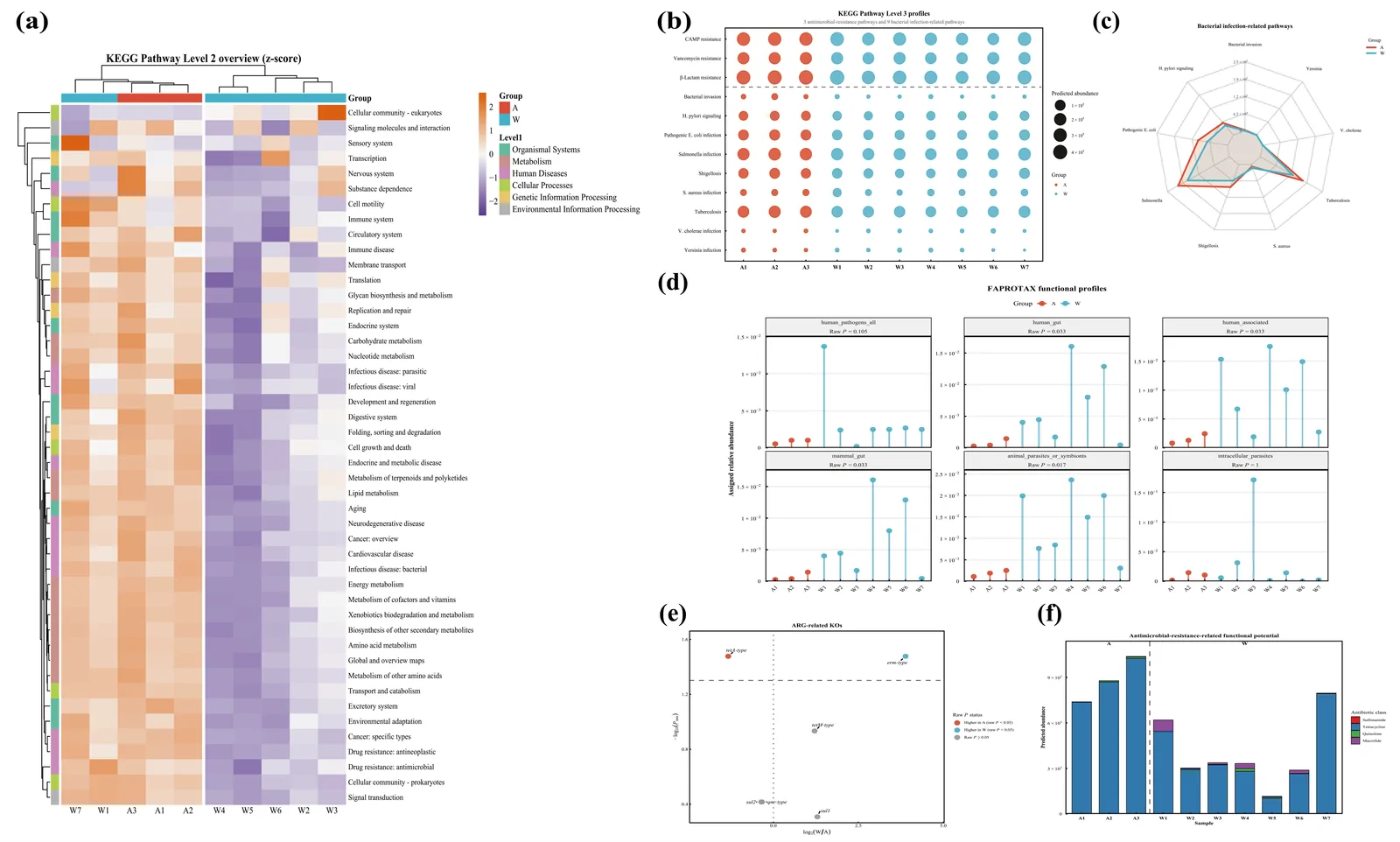
Multilevel functional prediction analysis of the bacterial communities of the two effluent types. (**a**) Z-score heatmap of 46 PICRUSt2-predicted KEGG Pathway Level 2 categories; (**b**) Bubble plot of 12 KEGG Pathway Level 3 pathways; (**c**) Radar chart showing the group means of nine bacterial infection-related pathways for Group A (red) and Group W (blue); (**d**) Taxon-based FAPROTAX assignments for six pathogen- and host-associated functional categories across the 10 samples; (**e**) Volcano plot comparing the predicted abundances of six ARG-related KOs between Group A and Group W. These KOs were labeled as *sul1*, *sul2*, *tetA*-type, *tetM*-type, *qnr*-type, and *erm*-type. Colors are based on unadjusted *p* values; (**f**) Sample-level stacked bar chart of the same six ARG-related KOs aggregated into four antibiotic classes. All PICRUSt2 and FAPROTAX results represent inferred functional potential rather than directly measured genes or activities. In (**e**), the vertical dotted line indicates log_2_(W/A) = 0, and the horizontal dashed line indicates the unadjusted *p* = 0.05 threshold. In (**f**), the vertical dashed line separates Group A from Group W samples.

The PICRUSt2-predicted KEGG Pathway Level 2 heatmap included 46 pathway categories, with the predicted abundance of each pathway standardized as a z-score (Figure 4a). Hierarchical clustering grouped all Group A samples with W1 and W7, whereas W2–W6 formed a second cluster. The predicted functional profiles showed partial differentiation between the two effluent types. Group A generally showed higher standardized scores for several basic metabolic and transport categories. These included membrane transport, carbohydrate metabolism, replication and repair, amino acid metabolism, and xenobiotics biodegradation and metabolism. By comparison, Group W displayed greater sample-level variation. In particular, W1 and W7 differed from the other Group W samples.

Several sample-specific patterns were observed. W3 showed a relatively high standardized score for cellular communities–eukaryotes, whereas W2 showed a relatively high score for signaling molecules and interaction. Within Group W, W1 and W7 showed relatively high scores for cell motility and for development and regeneration. Group A also generally showed higher standardized scores for several KEGG Level 2 categories labeled infectious disease and drug resistance. These KEGG categories represent shared predicted functional annotations and do not demonstrate pathogen occurrence, disease risk, or phenotypic antimicrobial resistance. Overall, the predicted functional profiles showed partial differentiation between the two effluent types and variation among individual samples.

#### 3.4.2. Predicted Resistance and Bacterial Infection-Related Pathways

The bubble plot shows the predicted abundances of 12 KEGG Level 3 pathways across the 10 samples (Figure 4b). These included three antimicrobial-resistance pathways and nine bacterial infection-related pathways. Cationic antimicrobial peptide resistance, vancomycin resistance, and β-lactam resistance showed higher mean predicted abundances in Group A. However, none of these three pathways differed significantly between the groups.

Among the nine bacterial infection-related pathways, seven showed higher mean predicted abundances in Group A. In contrast, *Staphylococcus aureus* infection and *Vibrio cholerae* infection showed higher mean predicted abundances in Group W. The radar chart further illustrates these group-level patterns (Figure 4c). The Wilcoxon rank-sum test yielded a raw *p* value of 0.033 for bacterial invasion of epithelial cells. However, this difference did not remain significant after BH correction (adjusted *p* = 0.267). Pathogenic *Escherichia coli* infection and tuberculosis each had a raw *p* value of 0.067. Excluding W5 produced a similar result for bacterial invasion of epithelial cells (raw *p* = 0.048; adjusted *p* = 0.381). These group-level patterns may partly reflect differences in bacterial community composition between the two effluent types.

#### 3.4.3. FAPROTAX-Assigned Host- and Pathogen-Associated Functions

Six host- and pathogen-associated functional categories were selected for further analysis: human pathogens, human gut, human-associated, mammal gut, animal parasites or symbionts, and intracellular parasites (Figure 4d). Four categories showed higher mean assigned relative abundances in Group W and raw *p* < 0.05. These were human gut, human-associated, mammal gut, and animal parasites or symbionts. The corresponding raw *p* values were 0.033, 0.033, 0.033, and 0.017, respectively. However, their BH-adjusted *p* values were 0.050, and none met the predefined adjusted *p* < 0.05 criterion. Animal parasites or symbionts had a mean assigned relative abundance of 0.014 in Group W and 0.002 in Group A. Sample-level relative abundances of all FAPROTAX-assigned categories are provided in Table S10A.

The intracellular parasites category showed a specific peak of 0.171 in W3. Its mean assigned relative abundance was 0.033 in Group W and 0.009 in Group A. The W3 value was approximately 18.7 times the Group A mean. This pattern coincided with high relative abundances of *Legionella* (16.7%), *Flavobacterium* (11.5%), and *Mycobacterium* (7.0%) in W3. Some species within these genera have been associated with opportunistic infections or host-associated environments. W3 received mixed discharges of poorly pretreated rural domestic sewage and compositionally complex industrial park wastewater, which may have increased influent heterogeneity. Together with the conventional A^2^/O process used at W3, these variable influent conditions may partly account for the relatively high abundances of Legionella and Mycobacterium observed in this sample. However, genus-level 16S data and FAPROTAX assignments cannot confirm pathogenic species, virulence, or cell viability. After W3 was excluded, the Group W mean for intracellular parasites decreased to 0.009 and was similar to the Group A mean of 0.009. The difference remained nonsignificant (adjusted *p* = 0.714), indicating that the higher Group W mean was mainly driven by W3. In the W3-excluded sensitivity analysis, human pathogens, human-associated, and animal parasites or symbionts remained higher in Group W (adjusted *p* = 0.050 for each). Complete statistics for the main and W3-excluded FAPROTAX analyses are provided in Table S10B. These sensitivity results were interpreted cautiously because of the small sample size.

For both the human-gut and mammal-gut categories, the mean assigned relative abundance was higher in Group W than in Group A (0.007 vs. 0.001). This pattern was consistent with the greater representation of enteric-associated microorganisms in several Group W samples. Overall, the FAPROTAX profiles showed both group-level and sample-specific variation, particularly in W3. These taxon-based assignments represent inferred ecological associations rather than direct evidence of pathogens, host origin, or functional activity.

#### 3.4.4. Antimicrobial-Resistance-Related Functional Potential

Six ARG-related KOs were selected from the PICRUSt2-predicted functional profiles to characterize antimicrobial-resistance-related functional potential. Selection was based on the KEGG Biomolecular Relations in Information Transmission and Expression antimicrobial resistance classification, including ko01504, reference tables br01553–br01557, and the br01600 signature-KO system (Table 3). Full main-analysis statistics for the six prespecified ARG-related KOs are provided in Table S8. The *tetA*-type KO showed a higher mean predicted abundance in Group A than in Group W (8653 vs. 3449). This difference had a raw *p* value of 0.033 and a Cliff’s delta of −0.905, calculated as W minus A. However, the difference did not remain significant after BH correction (adjusted *p* = 0.100). In contrast, the *erm*-type KO showed a higher mean predicted abundance in Group W than in Group A (221 vs. 14). Its raw *p* value was 0.033, and Cliff’s delta was 0.905. This difference also did not remain significant after BH correction (adjusted *p* = 0.100; Figure 4e).

The *tetM*-type and *sul1* KOs showed higher mean predicted abundances in Group W. The *sul2* and *qnr*-type KOs showed slightly higher mean predicted abundances in Group A. None of these four KOs differed significantly between the groups. The descriptive aggregate of the six selected ARG-related KOs was approximately 2.22-fold higher in Group A than in Group W (8850 vs. 3992; Figure 4f). The three selected antimicrobial-resistance pathways also showed higher mean predicted abundances in Group A, although none differed significantly between the groups. Because W5 had a high abundance-weighted NSTI value, a sensitivity analysis excluding W5 was conducted. The higher *tetA*-type prediction in Group A and the higher *erm*-type prediction in Group W remained directionally consistent. Both had a raw *p* value of 0.048 and an adjusted *p* value of 0.143. Detailed results of the W5-excluded ARG-related KO sensitivity analysis are provided in Table S7B. After W5 was excluded, the aggregate predicted abundance remained approximately 1.98-fold higher in Group A.

Overall, Group A showed higher predicted *tetA*-type and aggregate antimicrobial-resistance-related functional potential. Group W showed higher predicted *erm*-type functional potential. However, none of the six KO-level differences remained significant after BH correction. These results were therefore interpreted as exploratory predictions rather than direct measurements of ARG abundance or phenotypic antimicrobial resistance.

### 3.5. NSTI Evaluation and Cross-Study Concordance Analysis

The recalculated abundance-weighted NSTI values of the 10 samples ranged from 0.049 to 0.276, with a median of 0.072. Seven samples had abundance-weighted NSTI values below 0.10. Per-sample abundance-weighted NSTI values and group-level sensitivity summaries are provided in Tables S6 and S7A, respectively. A3 had the lowest value (0.049), whereas W5 had the highest value (0.276). The mean NSTI values were 0.064 in Group A and 0.127 in Group W. After W5 was excluded, the Group W mean decreased to 0.103. W5 was retained in the main analysis, and a sensitivity analysis excluding W5 was conducted. The directions of the tetA-type and erm-type results remained consistent, and no KO-level difference remained significant after BH correction. These results indicated variation among samples in the representation of the observed OTUs by available sequenced reference genomes. Cross-study concordance was assessed using datasets from three published studies [22,23,24]. These included qPCR, SmartChip, and metagenomic ARG profiles from reclaimed-water, WWTP–constructed wetland, and industrial mariculture systems. The comparison focused on the correspondence between detected ARGs and six PICRUSt2-predicted ARG-related KOs at the gene, ARG-family, or resistance-mechanism level. Different levels of correspondence were observed among the datasets (Table 4).

**Figure 5:**
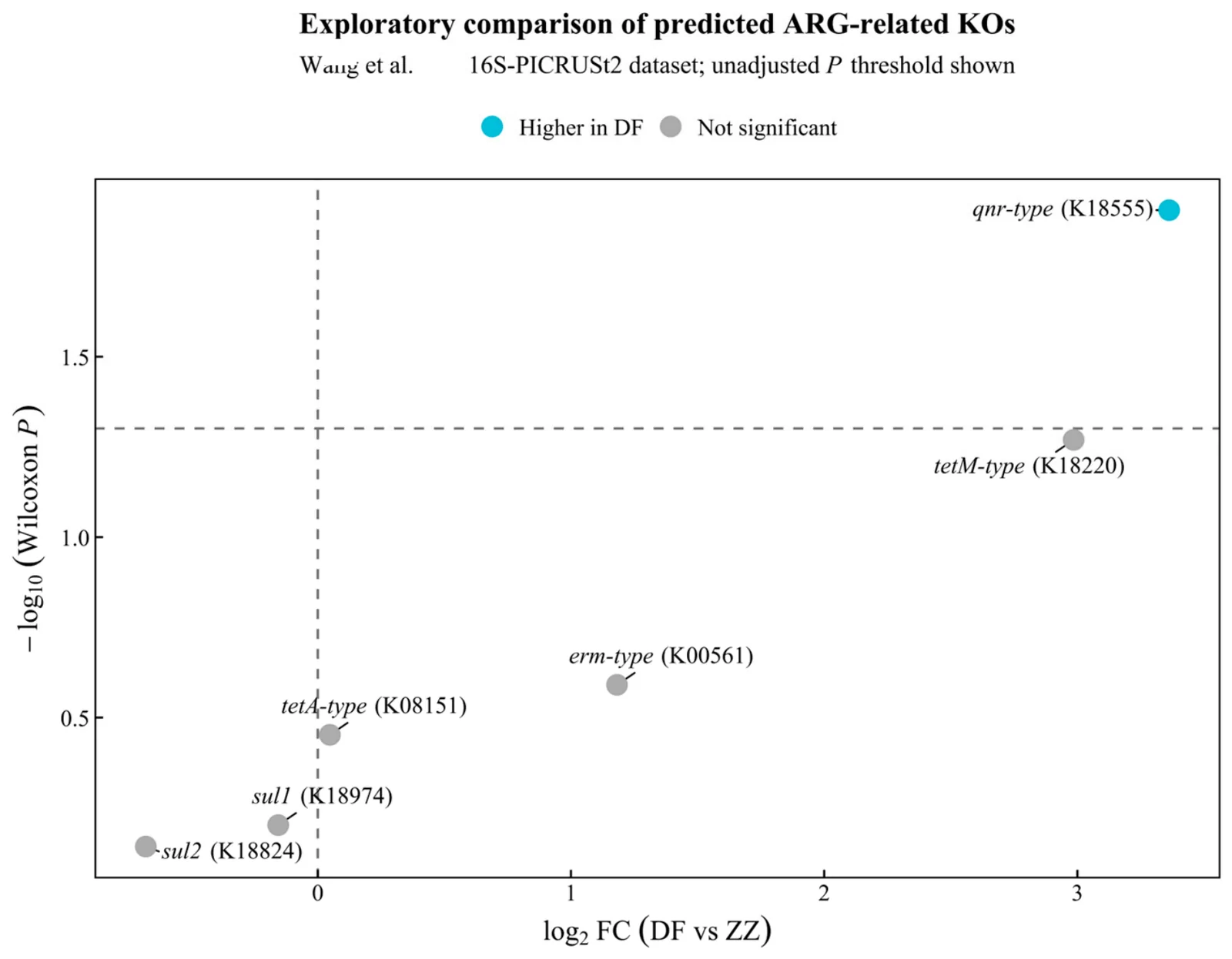
Exploratory comparison of six PICRUSt2-predicted ARG-related KOs in the Wang et al. [24] dataset. The volcano plot compares the two groups (DF and ZZ; labels retained from the original dataset). The vertical dashed line indicates log_2_FC (DF vs. ZZ) = 0, and the horizontal dashed line indicates the unadjusted *p* = 0.05 threshold.

Applying the same 16S-based workflow to the Wang et al. dataset predicted all six targets (Figure 5). Corresponding genes, ARG families, or resistance mechanisms were also identified in the metagenomic profiles [24]. K18974 and K18824 showed direct gene-level correspondence with *sul1* and *sul2*, respectively, in all three datasets. For K08151, *tetA* in the Wang et al. dataset provided direct correspondence. The detection of *tetB* and *tetG* in all three datasets provided additional correspondence at the tetracycline-efflux-mechanism level. K18220 showed direct correspondence with *tetM* in the Zhang et al. and Wang et al. datasets [23,24]. K18555 showed ARG-family-level correspondence with *qnr* genes detected in the Lu et al. and Wang et al. datasets [22,24]. For K00561, *ermF* and *ermT* were detected in the Lu et al. and Zhang et al. datasets [22,23], whereas *ermF* was detected in the Wang et al. dataset [24]. These genes encode *erm*-type 23S rRNA methyltransferases but are not exact gene-level matches to K00561. They therefore provided resistance-mechanism-level correspondence.

At the microbial community level, *Trichococcus* was the genus with the highest average abundance in this study and also the dominant genus in W6. It was associated with multiple ARGs in the networks reported by Lu et al. [22] and Zhang et al. [23]. In the reported WWTP networks [23], *Pseudarcobacter* was positively correlated with two ARG subtypes, and *Arcobacter* was involved in six positive ARG–genus correlations. These two genera were also characteristic of W4 and W5 in the present study. Wang et al. [24] identified Proteobacteria and Bacteroidetes as potential predominant hosts of ARGs in mariculture systems. Bacteroidetes corresponds to Bacteroidota in the SILVA nomenclature used in the present study, and ARGs attributed to this phylum were mainly associated with ribosomal-protection mechanisms. Collectively, the concordance observed at the gene, ARG-family, resistance-mechanism, and microbial-community levels provides complementary external context for interpreting the PICRUSt2-predicted ARG-related KOs.

## 4. Discussion

Integrating genus-level community composition, environmental associations, and functional predictions suggested distinct bacterial patterns in the two effluent types. The WHO 2024 BPPL provided context for interpreting genera of potential public-health relevance. Functional assignments and predictions were treated as complementary, hypothesis-generating evidence. Group A showed a relatively consistent community structure. *Pseudomonas* averaged 7.2% and reached 10.3% in A3. *Acinetobacter* averaged 8.7% and reached 22.0% in A1. Both genera include species–resistance combinations listed in the WHO 2024 BPPL. Carbapenem-resistant *Acinetobacter baumannii* is categorized as Critical priority, whereas carbapenem-resistant *Pseudomonas aeruginosa* is categorized as High priority. However, genus-level detection does not confirm these species or their resistance phenotypes. Similar aquaculture-related sources may have contributed to the apparent community similarity among Group A samples. This interpretation remains tentative because Group A included only three independent sites sampled at a single time point.

Group W showed greater descriptive sample-specific variation. W3 contained high relative abundances of *Legionella* (16.7%), *Flavobacterium* (11.5%), and *Mycobacterium* (7.0%). These genera include species associated with opportunistic infections or engineered water systems [37,38]. The genus *Mycobacterium* includes rifampicin-resistant *Mycobacterium tuberculosis*, which is listed in the WHO 2024 BPPL. However, the present genus-level results do not demonstrate the occurrence of *M. tuberculosis*, *Legionella pneumophila*, or other pathogenic species. The intracellular-parasites FAPROTAX category also peaked in W3. After W3 was excluded, its mean assigned relative abundance was similar between the groups, indicating that this pattern was mainly driven by a single sample. *Pseudarcobacter* and *Arcobacter* were characteristic of W4 and W5, suggesting possible enteric or wastewater-related influences [17,28,29,34,35]. W2 lacked a single dominant genus and contained numerous low-abundance taxa. These patterns indicated substantial taxonomic variation among WWTP effluents. However, PERMDISP did not detect a significant difference in within-group dispersion between Groups A and W.

The environmental analyses provided limited evidence for specific environmental associations. The overall CCA model was not significant (*p* = 0.803), and the CCA results of this study were interpreted as exploratory. Among 400 genus–factor correlations, only the negative association between *Sphingorhabdus* and COD remained significant after BH correction (Spearman’s ρ = −0.952, adjusted *p* = 0.009). This relationship may partly reflect group-level differences in both COD and relative abundance.

Descriptive differences were observed in antimicrobial-resistance-related functional potential. The aggregate predicted abundance of the six selected ARG-related KOs was approximately 2.22-fold higher in Group A than in Group W. This aggregate pattern was largely driven by the *tetA*-type KO, which was higher in Group A (8653 vs. 3449; raw *p* = 0.033). In contrast, the *erm*-type KO was approximately 15.8-fold higher in Group W (221 vs. 14; raw *p* = 0.033). Neither difference remained significant after BH correction (adjusted *p* = 0.100 for both). After W5 was excluded, the aggregate prediction remained 1.98-fold higher in Group A, and the directions of the *tetA*-type and *erm*-type patterns remained unchanged. Nevertheless, no KO-level difference remained significant after BH correction. These patterns may be related to differences in microbial composition and wastewater sources.

The three external datasets provided contextual evidence for the 16S-based functional predictions [22,23,24]. When the same workflow was applied to the Wang et al. dataset, all six predicted targets showed correspondence with metagenomic results at the gene, ARG-family, or resistance-mechanism level [24]. Published studies also reported potential associations involving several prominent genera identified here, including *Trichococcus*, *Pseudarcobacter*, and *Arcobacter*.

The recalculated abundance-weighted NSTI values ranged from 0.049 to 0.276, and seven samples had values below 0.10. W5 had the highest value, indicating lower reference-genome support than the other samples. Sensitivity analyses excluding W5 retained the main directional patterns but did not identify any BH-significant KO-level difference. The main comparison of the FAPROTAX human-pathogens category was also nonsignificant after BH correction (adjusted *p* = 0.126). PICRUSt2 and FAPROTAX outputs were therefore used as complementary functional evidence to support the interpretation of genus-level community patterns derived from 16S rRNA gene sequencing [21].

This study has limitations, as 16S rRNA-based genus-level analysis cannot distinguish strain-specific pathogenic and resistant species, and predicted functions cannot directly verify the actual presence of pathogens, ARGs, or phenotypic resistance. Even so, the low-cost, high-throughput 16S-based framework is highly suitable for large-scale and routine water monitoring. Future studies will incorporate continuous sampling, metagenomic sequencing, targeted ARG quantification and culture-based identification to validate the temporal stability and health implications of the observed patterns.

## 5. Conclusions

This study applied a 16S rRNA-based comparative screening framework and identified distinct bacterial community configurations in aquaculture and WWTP effluents from the Mi River Basin. Group A shared several abundant genera, including *Acinetobacter*, *Limnohabitans*, and *Pseudomonas*. Their occurrence highlights targets for species-level confirmation under the WHO 2024 BPPL framework. Group W showed marked site-specific patterns. W3 contained high relative abundances of *Legionella*, *Flavobacterium*, and *Mycobacterium*, whereas *Pseudarcobacter* and *Arcobacter* characterized W4 and W5. Environmental analysis identified the negative association between *Sphingorhabdus* and COD as the only genus–factor relationship that remained significant after BH correction. Functional prediction also revealed contrasting directional patterns between the two effluent types. The aggregate predicted abundance of six ARG-related KOs was 2.22-fold higher in Group A, largely because of the *tetA*-type KO. In contrast, the *erm*-type KO showed a higher mean predicted abundance in Group W. Although these KO-level differences did not remain significant after BH correction, their directions were retained after excluding W5. Cross-study correspondence at the gene, ARG-family, and resistance-mechanism levels further identified these KOs as useful targets for confirmatory analysis. Together, these findings support a tiered monitoring strategy for effluent management. Since this study relies on 16S rRNA gene-based genus-level analysis and functional prediction, further studies combining continuous sampling, metagenomic sequencing, ARG quantification, and culture-based assays are required to validate the temporal stability and health risks of the observed microbial patterns.

## Figures and Tables

**Table 1:** Comparison of physicochemical water-quality parameters between aquaculture effluent (Group A) and WWTP effluent (Group W).

Parameter	Group A (Mean ± SD)	Group W (Mean ± SD)	A vs. W	Wilcoxon W	Cliff’s Delta (A − W)	Raw *p*	BH-Adjusted *p*
T (°C)	16.83 ± 0.86	15.31 ± 2.71	A > W	15	0.429	0.383	0.517
pH	8.23 ± 0.12	8.11 ± 0.33	A > W	14	0.333	0.493	0.517
DO (mg/L)	8.15 ± 0.97	9.43 ± 0.57	W > A	2	−0.810	0.067	0.178
Sal (ppm)	460.33 ± 196.41	2631.43 ± 1606.74	W > A	0	−1.000	0.017	0.067
COD (mg/L)	11.33 ± 5.69	33.71 ± 12.07	W > A	0	−1.000	0.017	0.067
TP (mg/L)	0.28 ± 0.23	0.15 ± 0.12	A > W	14.5	0.381	0.422	0.517
TN (mg/L)	22.72 ± 21.62	10.13 ± 4.96	A > W	14.5	0.381	0.424	0.517
NH_3_-N (mg/L)	0.67 ± 0.90	0.90 ± 0.92	W > A	7	−0.333	0.517	0.517

Values are presented as mean ± SD (Group A, *n* = 3; Group W, *n* = 7). Raw *p* values were obtained using two-sided Wilcoxon rank-sum tests. BH correction was applied across all eight parameters. Cliff’s delta was calculated as Group A minus Group W; negative values indicate higher values in Group W. No parameter remained significant after BH correction.

**Table 2:** Dominant genera in each Group W sample after removal of chloroplast and mitochondrial sequences (relative abundances ≥ 5%).

Sample	Dominant Genera (Relative Abundance ≥ 10%)	Other Genera (5% ≤ Relative Abundance < 10%)
W1	*Achromobacter* (11.5%)	*Massilia* (8.1%); *Acinetobacter* (5.9%); *Turicibacter* (5.7%); *Flavobacterium* (5.6%); *Bacillus* (5.0%)
W2	No genus with relative abundance ≥ 10%	No genus with relative abundance ≥ 5%
W3	*Legionella* (16.7%); *Flavobacterium* (11.5%)	norank_o__Candidatus_Kaiserbacteria (7.4%); *Trichococcus* (7.2%); *Mycobacterium* (7.0%)
W4	*Pseudarcobacter* (19.9%); *Trichococcus* (14.4%)	*Arcobacter* (9.3%); *Acinetobacter* (5.1%)
W5	*Pseudarcobacter* (14.4%)	*Arcobacter* (7.3%); norank_o__Candidatus_Campbellbacteria (6.9%); *Trichococcus* (6.4%); norank_o__Candidatus_Peregrinibacteria (5.2%)
W6	*Trichococcus* (41.7%)	All remaining genera < 5%
W7	*Rhodoferax* (26.7%); *Flavobacterium* (13.7%); Candidatus Obscuribacter (10.5%)	*Massilia* (9.3%); norank_f__Obscuribacteraceae (7.6%)

**Table 3:** Six ARG-related KOs selected for predicted functional analysis.

KO	Functional Label	Antibiotic Classes	Source (Literature Citation)
K18974	*sul1*	Sulfonamides	[12,13,14]
K18824	*sul2*	Sulfonamides	[12,14]
K08151	*tetA*-type	Tetracyclines	[12,14]
K18220	*tetM*-type	Tetracyclines	[12,13,14]
K18555	*qnr*-type	Quinolones	[12,14]
K00561	*erm*-type	Macrolides	[12,13,14]

**Table 4:** Cross-study correspondence between six PICRUSt2-predicted ARG-related KOs and ARGs detected in datasets associated with published studies.

Predicted KO	KEGG Annotation	Corresponding ARG Detections	Study-Associated Datasets	Level of Correspondence
K18974	*sul1*; dihydropteroate synthase type 1	*sul1*	[22,23,24]	Direct gene-level correspondence
K18824	*sul2*; dihydropteroate synthase type 2	*sul2*	[22,23,24]	Direct gene-level correspondence
K08151	*tetA*-type; MFS tetracycline resistance protein	*tetA*, *tetB*, and *tetG*	*tetA*: [24]; *tetB* and *tetG*: [22,23,24]	Direct gene-level correspondence through *tetA*; tetracycline-efflux-mechanism correspondence through *tetB* and *tetG*.
K18220	*tetM*-type; ribosomal protection tetracycline resistance protein	*tetM*	[23,24]	Direct gene-level correspondence through *tetM*
K18555	*qnr*-type; fluoroquinolone resistance protein	*qnrA*, *qnrB*, *qnrS*	*qnrA*, *qnrB*, and *qnrS*: [22]; *qnrS*: [24]	ARG-family-level correspondence
K00561	*erm*-type; 23S rRNA (adenine-N6)-dimethyltransferase	*ermF*, *ermT*	*ermF* and *ermT*: [22,23]; *ermF*: [24]	Resistance-mechanism-level correspondence

## Data Availability

The raw 16S rRNA gene amplicon sequencing data generated in this study have been deposited in the NCBI Sequence Read Archive (SRA) under BioProject accession PRJNA1531991. The associated SRA run accession numbers are SRR40760104–SRR40760113. Other original contributions presented in this study are included in the article/Supplementary Materials. Further inquiries can be directed to the corresponding authors.

## Supplementary Materials

The following supporting information can be downloaded at: https://www.mdpi.com/article/10.3390/toxics14100852/s1: Table S1A: Sample metadata for the 10 independent effluent samples; Table S1B: Mean relative abundances of major bacterial phyla in aquaculture effluent (Group A) and WWTP effluent (Group W); Table S2: Alpha-diversity indices and Wilcoxon rank-sum test results; Table S3: PERMANOVA and PERMDISP results based on Bray–Curtis dissimilarities; Table S4A: Inertia summary for the exploratory canonical correspondence analysis; Table S4B: Overall and axis-level permutation tests for the exploratory canonical correspondence analysis; Table S4C: Variance inflation factors for the environmental variables included in the exploratory CCA; Table S5: Full genus–environment Spearman correlation results; Table S6: Recalculated abundance-weighted NSTI values for the 10 samples; Table S7A: Group-level NSTI summary for the main and W5-excluded sensitivity analyses; Table S7B: Sensitivity analysis of the six ARG-related KOs after excluding W5; Table S8: Main-analysis statistics for the six prespecified ARG-related KOs; Table S9: PICRUSt2-predicted abundance of all KEGG Orthology entries across the 10 samples; Table S10A: Sample-level relative abundance of all FAPROTAX-assigned functional categories; Table S10B: Group comparisons of the six selected FAPROTAX categories in the main and W3-excluded sensitivity analyses.

## Abbreviations

The following abbreviations are used in this manuscript:

ARG	Antibiotic resistance gene
ARG-related KO	KEGG Orthology associated with antibiotic-resistance-related functions
ARB	Antibiotic-resistant bacterium
WWTP	Wastewater treatment plant
BPPL	Bacterial Priority Pathogens List
WHO	World Health Organization
T	Temperature
DO	Dissolved oxygen
Sal	Salinity
COD	Chemical oxygen demand
NH_3_-N	Ammonia nitrogen
TP	Total phosphorus
TN	Total nitrogen
NSTI	Nearest Sequenced Taxon Index
OTU	Operational taxonomic unit
KEGG	Kyoto Encyclopedia of Genes and Genomes
KO	KEGG Orthology
PICRUSt2	Phylogenetic Investigation of Communities by Reconstruction of Unobserved States 2
FAPROTAX	Functional Annotation of Prokaryotic Taxa
A^2^/O	Anaerobic-anoxic-oxic
NMDS	Non-metric multidimensional scaling
CCA	Canonical correspondence analysis
PERMANOVA	Permutational multivariate analysis of variance
ACE	Abundance-based coverage estimator
SD	Standard deviation
MFS	Major facilitator superfamily
BH	Benjamini–Hochberg
PERMDISP	Permutational analysis of multivariate dispersions
qPCR	Quantitative polymerase chain reaction
RDP	Ribosomal Database Project
